# Survival after extracorporeal cardiopulmonary resuscitation for adolescent arrhythmogenic arrest: ECPella (extracorporeal membrane oxygenation with Impella^®^)—a case report

**DOI:** 10.1093/ehjcr/ytae581

**Published:** 2024-11-07

**Authors:** Anouska Lerner, Ajay Desai, Richard Trimlett, Janice Till, Amy Chan-Dominy

**Affiliations:** Department of Paediatric Cardiology, Royal Brompton Hospital, Sydney Street, London SW3 6NP, UK; PICU, Level 4, Royal Brompton Hospital, Sydney Street, London SW3 6NP, UK; AICU, Level 5, Royal Brompton Hospital, Sydney Street, London SW3 6NP, UK; Department of Paediatric Cardiology, Royal Brompton Hospital, Sydney Street, London SW3 6NP, UK; PICU, Level 4, Royal Brompton Hospital, Sydney Street, London SW3 6NP, UK

**Keywords:** ECPella, ECMO, Sudden arrhythmic cardiac arrest, Adolescent, Case Report

## Abstract

**Background:**

The combined therapy with venoarterial extracorporeal membrane oxygenation and Impella (ECPella) has been shown to improve survival in acute cardiogenic shock (CS) in adult patient. Only three paediatric cases have been reported in a multicentre study.

**Case summary:**

We present our case, the first described to our knowledge in the UK, of a 15-year-old adolescent of Afro-Caribbean descent, weight 75 kg, who received extracorporeal cardiopulmonary resuscitation (E-CPR) and ECPella implantation. The patient suffered a sudden cardiac arrest at home; his mother called for an ambulance that arrived within 10 min and commenced life support with a LUCAS device. He received three doses of adrenaline and three desynchronized shocks for an underlying rhythm of ventricular fibrillation (VF) after which return of spontaneous circulation was achieved. He was then transferred to his local hospital where he had another VF arrest with successful cardioversion and was then transferred to our institution where on arrival he had another VF arrest and received E-CPR and ECPella implantation under the institutional adult shock programme within 3 h of in-hospital cardiac arrest. Following weaning from ECPella, the patient underwent cardiac and brain magnetic resonance imaging and serial echocardiograms with complete recovery of ventricular function. After implantation of cardiac defibrillator, he was discharged home without neurological sequelae. He remains asymptomatic from a cardiac perspective, with a normal cardiac examination and with no neurological sequelae at 2-year follow-up.

**Discussion:**

This is the first case description of ECPella use in a child in the UK and highlighted the importance of timely institution of E-CPR on survival benefit in fatal CS. The outcome success of post-resuscitation ECPella strategy in this adolescent was through collaborative interprofessional engagement of multiple supra-specialists within acute cardiology and critical care across paediatric and adult services and alignment with the institutional adult shock programme.

**Learning points**

- Beyond cardiopulmonary resuscitation (CPR) and advanced life support, survival from an unexpected sudden cardiac arrest in the young relies on access and capacity to receive extracorporeal life support.
- High-quality extracorporeal CPR is a complex intervention that provides a life-sustaining bridge to therapy, diagnostics, and recovery for intractable arrhythmias degrading to refractory cardiogenic shock.
- Goals of peri-resuscitation care with extra cardiac life support include haemodynamic-directed interventions to optimize myocardial recovery and prevention of secondary neurological injury by minimizing duration of ECMO and anticipating scenario destination.
- Innovative use of established technologies in different populations has implications for training and governance, by collaborative responsibilities of adult and paediatric cross-specialty teams towards model of care for young adolescents taking respectful consideration of anthropometry limitations, age-based physiology, and pathology.

## Introduction

Sudden arrhythmic cardiac arrest in the young is rare, but a significant cause of sudden cardiac death (SCD). The current literature^1^ shows after 40 min of standard cardiopulmonary resuscitation (CPR), less than 1% of adults will achieve return of spontaneous circulation (ROSC) and favourable neurological outcome. The recent ARREST trial^2^ showed the benefit of utilizing extracorporeal membrane oxygenation (ECMO) in refractory cardiac arrest, with 43% of cases in the study receiving extracorporeal CPR (E-CPR) achieving survival to discharge with favourable neurological outcome vs. only 7% receiving standard adult life support care. Unfortunately, the equivalent data sets in paediatrics are lacking.

The combination of ECMO with an Impella device to offload the left ventricle (LV) has had some success in the adult population,^3^ but its use in the paediatric population so far has been limited. At present, there is no randomized control trial to support the approach of Impella in E-CPR cases, in adults or children. We believe this case shows the important of multidisciplinary team working and expertise sharing between adult and paediatric cardiologists and intensivists to facilitate a good outcome in the first successful use of ECPella therapy in an adolescent in the UK.

## Summary figure

**Figure:**
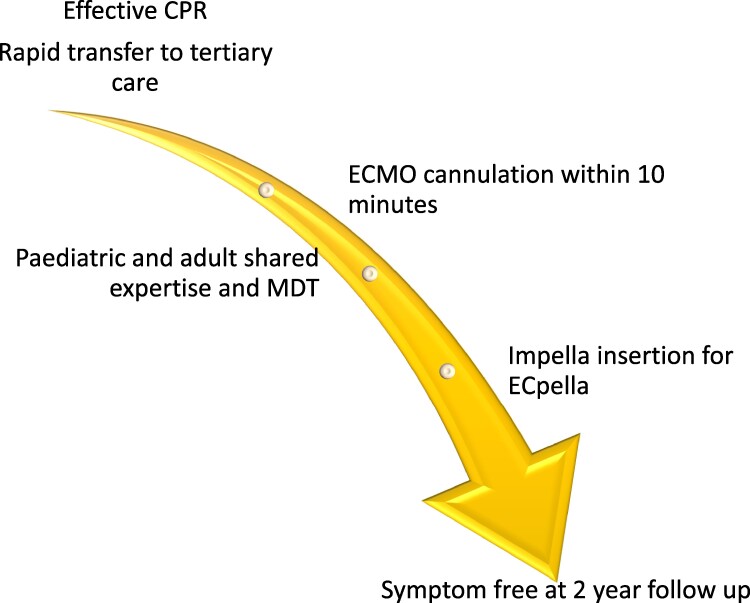


## Case summary

**Figure 1:**
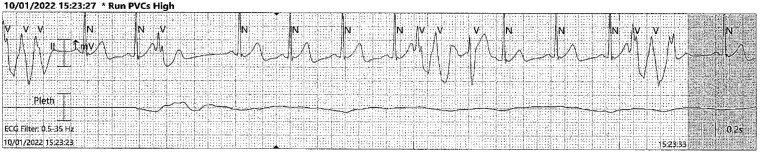
Rhythm strip (1) from telemetry showing a similar morphology ventricular ectopic beat triggering ventricular fibrillation repeatedly.

**Figure 2:**
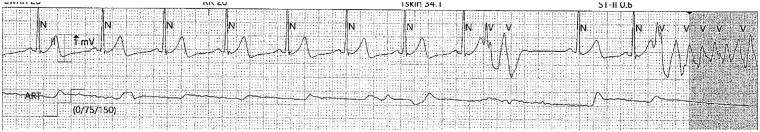
Rhythm strip (2) from telemetry again showing ventricular ectopic beat triggering an episode of ventricular fibrillation.

An 15-year-old adolescent athletic schoolboy of Afro-Caribbean descent (75 kg, body surface area 2.0 m^2^) with no previous healthcare needs had a sudden collapse at home and was immediately attended to by his mother. There was no family history of SCD in the family, nor any history of cardiac symptoms such as palpitations or syncope either in the patient, siblings, or parents. Basic life support was initiated, within 10 min paramedics arrived, continued CPR with a LUCAS device and three cycles of adrenaline. Ventricular fibrillation (VF) was cardioverted on the third attempt of direct current cardioversion with sinus rhythm and ROSC. On arrival at local hospital, he was intubated. He had a further cardiac arrest with ROSC, and peripheral adrenaline was started at 0.05 mcg/kg/min, and he was transferred to our tertiary paediatric cardiorespiratory intensive care unit (PICU) for further management (*Figures 1* and *2*).

Examination at admission to our PICU was that of a critically unwell child with poor peripheral perfusion and weak central and peripheral pulses. He was intubated and ventilated and on peripheral low-dose adrenaline at 0.05 mcg/kg/min. Cardiovascular examination showed normal heart sounds and no murmurs. Chest sounds were clear with good air entry and no crepitations, abdomen was soft, liver edge was not palpable, and there was no skin rash.

Soon after admission, his electrocardiogram (ECG) showed frequent ventricular ectopics, which appeared to trigger VF and cardiac arrest for which defibrillation with 200 J was given with ROSC. Amiodarone loading dose was given of 25 mcg/kg/min. Echocardiogram showed poor biventricular function. Admission arterial blood gas pH was 7.34 and lactate 3.7 mmol/L. Extracorporeal CPR protocol was activated for a repeat VF arrest, and venoarterial ECMO (VA-ECMO) was deployed at the bedside with percutaneous femoral cannulation using 25 Fr Biomedicus multistage cannula in left femoral vein and 19 Fr Biomedicus return cannula in left femoral artery. Time to cannulation was 10 min. Flow of 3.55 LPM on CardioHelp (Getinge) system was established.

Echocardiogram following cannulation showed severe biventricular dysfunction; the heart was however structurally normal with no valvar anomalies, prolapse, or regurgitation. Patient was transferred to cardiac catheter laboratory for further assessment. Emergency cardiac catheter study demonstrated normal coronary arteries; LV end-diastolic pressure was 22 mmHg indicating significant diastolic dysfunction.

**Figure 3:**
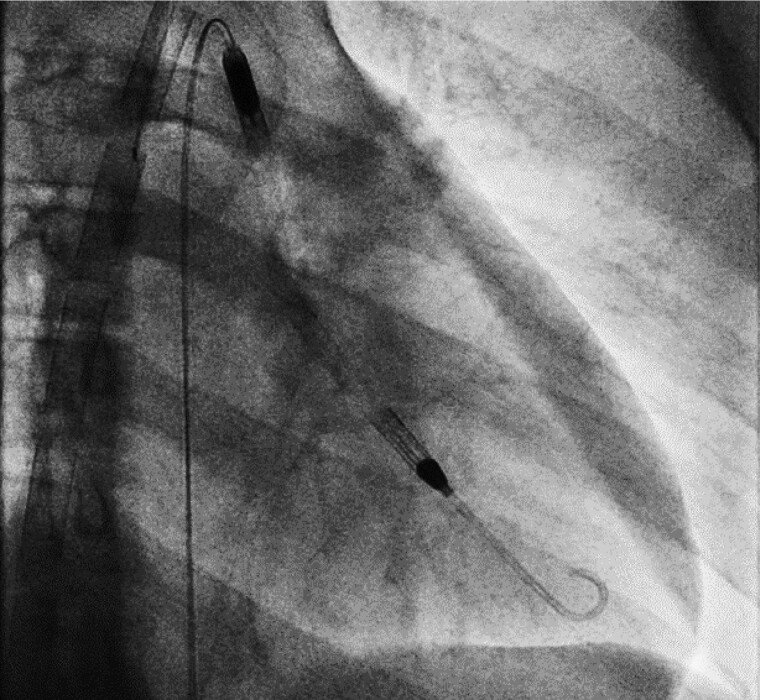
Fluoroscopy-guided placement of Impella device in the left ventricle.

A further VF cardiac arrest occurred whilst on ECMO in the cardiac catheter lab was terminated with 1 shock. On review of the available ECGs from the local hospital, there was initial sinus rhythm with no bundle branch block and a slightly prolonged QTc of 465 ms in the context of post-cardiac arrest and shock. On arrival at our institution, recurrent VF appeared to be triggered by close coupled ventricular ectopic beats (*Figure 3*). Unfortunately, a 12-lead ECG at this time failed to capture the ventricular ectopics, but they looked monomorphic on telemetry.

**Figure 4:**
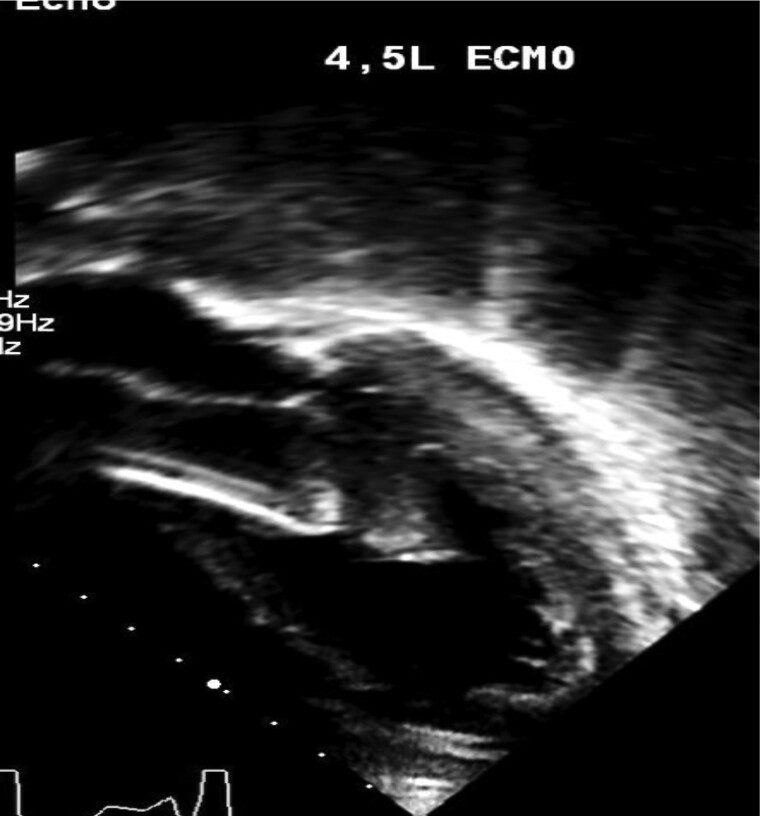
Still echocardiogram image showing Impella device in the left ventricle.

**Figure 5:**
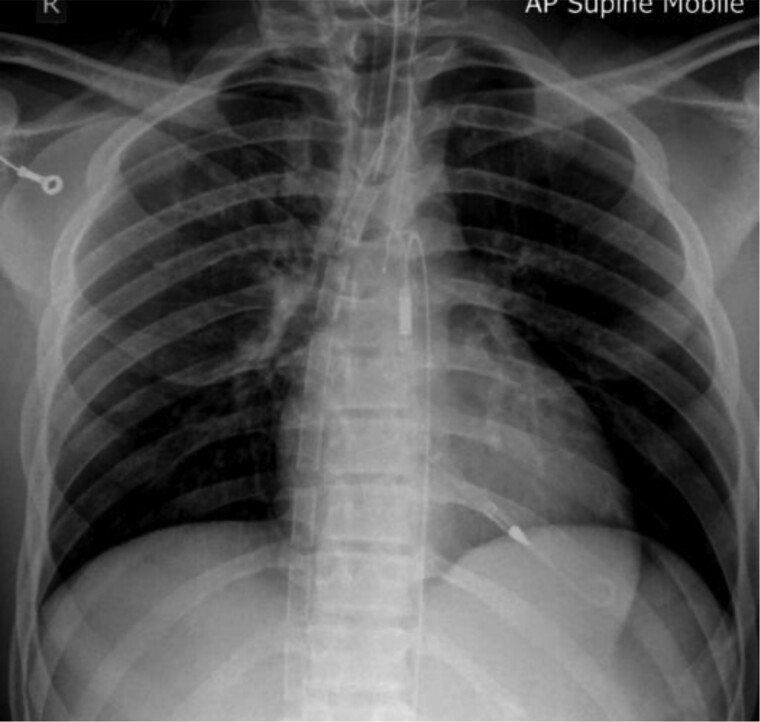
Chest radiograph showing extracorporeal membrane oxygenation cannula position and Impella device.

Emergency multidisciplinary meeting convened at the cardiac catheter laboratory and discussed optimal strategy for LV unloading. Given the goal was to mitigate LV pressure and volume injury in context of ectopic burden with minimal ventricular ejection, consensus was to proceed with percutaneous ventricular assist device. Based on patient’s LV cavity dimension, the adult interventional cardiologist inserted Impella® CP (Cardiac Power, Abiomed, Inc.) device via right femoral artery, with no peri-procedural complications (*Figures 3–5*). Overall management of patient with combination of peripheral VA-ECMO plus Impella (ECPella) pump support was provided by both adult and paediatric ECMO specialists.

Following insertion of the Impella device, there were no further documented ectopics or VF, and as such, quinidine was not felt necessary and ablation of possible triggering ventricular ectopics not possible or required. It is not possible to discern whether LV offloading with ECPella or amiodarone or the combination of both was the reason for cessation of activation of the ectopics that were triggering during this VF storm.

The patient’s cardiac function improved on ECPella support and amiodarone was continued. Patient was successfully weaned from ECPella after 91 h.

Explantation of ECMO and Impella was uneventful. The patient was extubated 24 h after decannulation. Echocardiography demonstrated normal ejection fraction. Cardiovascular magnetic resonance imaging (MRI) showed normal biventricular volumes and function, with no evidence of myocardial oedema/inflammation, infarction, or criteria for arrhythmogenic right ventricle cardiomyopathy. Brain MRI revealed no evidence of stroke or ischaemia. Genetic studies (R138 panel, the recommended panel for unexplained cardiac arrest as per the national recognized NHS England expert group consensus and national genetics directory) were normal. Neither resting nor exercise ECG showed evidence of long QT nor Brugada syndrome; his case was discussed at the arrhythmia and channelopathy multidisciplinary conference. With working diagnosis of idiopathic VF, a single-chamber implantable cardioverter-defibrillator (ICD) with DF-4 Abbott lead connected to Gallant single-chamber device was inserted in case of any further VF arrests. An S-ICD was not suitable in this case as screening for S-ICD in this patient failed in all three vectors secondary to left bundle branch block which developed during Impella implantation and did not resolve following removal and ECMO decannulation.

On Day 25, the patient was discharged home on amiodarone, with no end-organ complications. He underwent phased return to school by 8 weeks. He is being followed up in our ECMO recovery clinic as well as our electrophysiology clinic. Since discharge, he has not had any palpitations, and his cardiac examination remains normal. He has had three appropriate shocks for further VF, all of which were successful. He remains on amiodarone 200 mg OD and bisoprolol 5 mg OD which was increased from 2.5 mg following an episode of syncope. Echocardiogram shows preserved biventricular function, no functional impairment from his RV pacing lead. Electrocardiogram shows sinus rhythm at 56 b.p.m. with a QRS duration of 92 ms. His most recent device check shows ventricular sensing at 60 b.p.m. with a backup rate of 40 b.p.m., satisfactory battery, and lead measurements with a remaining battery life of 87 months. He has not had any syncope of ICD shocks for the last 6 months on current medication doses. He remains in education and with no discernible neurological deficit. All cardiomyopathy testing including genetics has been negative and as such has been diagnosed as idiopathic VF.

## Discussion

Although outcomes of conventional CPR (C-CPR) have improved over time, only 39–58% of patients in whom ROSC survive to hospital discharge.^4^ A secondary analysis of multicentre cluster randomized trial showed that in paediatric in-hospital cardiac arrest, medical cardiac patients had lower odds of survival with favourable neurologic outcomes compared with non-cardiac and surgical cardiac patients.^5^

Extracorporeal CPR is defined by the Extracorporeal Life Support Organization (ELSO) as VA-ECMO which is instituted during CCPR, delivered with manual or mechanical compressions, or within 20 min of the ROSC without ongoing compressions.^6^

Extracorporeal CPR may result in better outcomes compared to C-CPR.^1,2^ It provides adequate systemic oxygen delivery including the brain, kidneys, and intestines preventing secondary organ damage after a period of low cardiac output and hypoxia. Prompt deployment of E-CPR allows clinicians to reduce or stop vasoactive drugs, thereby reducing myocardial oxygen demand whilst providing adequate coronary perfusion (myocardial rest) allowing recovery. In addition, ECMO makes it possible to regulate the temperature of the patient, mitigating secondary damage due to hyperthermia that may follow cardiac arrest.^7^

In a study by Lasa *et al.*^8^ that compared 591 E-CPR patients with 3165 C-CPR patients (C-CPR > 10 min) using the American Heart Association Get with the Guidelines Registry (GWTG-R), after adjusting for covariates, patients receiving E-CPR had higher odds of survival to discharge [odds ratio (OR) 2.80, 95% confidence interval (CI) 2.13–3.69, *P* < 0.001] and survival with favourable neurologic outcome (OR 2.64, 95% CI 1.91–3.64, *P* < 0.001) compared to patients who received C-CPR. An outcome that has been replicated by the ARREST study^2^ in the USA and the PRAGUE-OHCA study^3^ within Europe.

Venoarterial ECMO comes at a cost of increased LV afterload leading to LV distension, increased left atrial pressures, and pulmonary oedema. Left ventricle unloading can be achieved using several strategies, and each approach has its own advantages and disadvantages.^9^ There is paucity of data to guide which patients supported with VA-ECMO may benefit from LV unloading.

Use of unloading strategies or devices can be associated with significant bleeding and vascular complications, haemolysis, and coagulation disorders in patients already prone to coagulopathy and systemic inflammatory response syndrome due to cardiac arrest. The use of additional mechanical circulatory support for unloading also adds practical complexity, not only in managing device settings but also for anticoagulation, monitoring vascular access sites, and positioning of patient. Therefore, one must balance risks against the potential benefits.

There are several ways to unload the LV, and these include (a) percutaneous catheter in LV, left atrium, or pulmonary artery and connected to drainage cannula of ECMO circuit; (b) percutaneous atrial septostomy; (c) intra-aortic balloon pump; and (d) percutaneous LV assist device (Impella).

An international multicentre cohort study showed LV unloading is associated with lower mortality in patients with cardiogenic shock treated with VA-ECMO, despite increased rates of severe bleeding and haemolysis.^10^

Use of Impella in the setting of ECMO or ECPR is commonly referred to as ECMELLA or ECPella. ECPella has been shown to reduce pulmonary capillary wedge pressure and reduce LV dimensions.^11^ Use of Impella has gained popularity in the adult setting to reduce ventricular work in cardiogenic shock patients treated with VA-ECMO with reported low mortality.^12^ Active LV unloading strategy with Impella provides a chance to increase LV recovery and also enables faster ECMO weaning, thus decreasing complication risks.^13^

Reported experience of ECPella is rare in the paediatric population.^12^ Use of Impella as a miniaturized catheter-based intravascular blood pump for single-agent therapy has been described in children, though rarely.^12^ Dimas *et al.*^12^ report the largest experience with 39 Impella implantations in patients younger than 21 years of age over a 6-year period, with six patients ≤10 years and ≤30 kg. Cardiogenic shock was the indication in 72%; 30-day mortality was 32%.

In the adult population, ECPella is associated with significant risk of bleeding, haemolysis, limb ischaemia, and need for continuous veno-venous haemofiltration compared to VA-ECMO alone.^13–15^ Risk profiles in the paediatric study were similar to Impella in adults.^11,12,16^ In the paediatric-adolescent cohort on ECPella support, incidence of major adverse events was 20%.

Regarding antiarrhythmic management, this patient presented with a VF arrest. It is our practice to use amiodarone acutely when the underlying aetiology of an arrhythmia is unclear as it is a comprehensive drug for arrhythmias. We then refine medication choice depending on what we discover to be the aetiology. So far, we have been unable to identify the underlying aetiology in this case. Therefore, our patient falls into the category of ‘idiopathic VF’.

In the acute setting, our patient was loaded with intravenous amiodarone at 25 mg/h for 4 h followed by maintenance dose of 15 mg/h (titration range 5–25 mg/h). Following decannulation, we converted to oral maintenance dose of 200 mg thrice daily. Our usual practice is to wean off amiodarone either in in-patient setting or in out-patient setting and replace with more targeted drug therapy. We added bisoprolol 2.5 mg, oral, once daily, in our patient at the time of discharge. Bisoprolol is very useful in a young person with an ICD as one of the commonest complications to having an ICD is an inappropriate shock when the ICD misinterprets ordinary sinus tachycardia as a rhythm that requires shocking—so it is a good practice to introduce and maintain a young patient with an ICD on bisoprolol.

In the meantime, our patient has had repeated VF. Happily, his ICD has detected and shocked appropriately. That has rather obscured the usual pathway. At the most recent follow-up, patient is on oral amiodarone, 200 mg once daily, and oral bisoprolol, 5 mg once daily. We think amiodarone will not be able to stop further episodes of VF occurring, and therefore, we are planning to wean and stop.

We believe this is the first case of successful peri-resuscitation treatment for sudden arrhythmic cardiac arrest and VF storm with insertion of Impella device in the UK. The collaborative effort between adult and paediatric specialists enabled cross-expertise from adult shock programme to extend the outcome benefits of ECPR.

## Data Availability

We the authors believe all relevant data to the case to be included in the above case report; however, the authors can be contacted if anyone would like further information.

## Lead author biography

Dr Anouska Lerner, paediatric cardiology registrar in London with an interest in structural heart disease, echocardiography, and foetal cardiology.

## Notes

**Consent:** Patient consent for this case report was obtained from the patient as well as next of kin in writing in keeping with the COPE guidelines. The family felt it was important for the wider medical community to be able to learn from this case.

**Funding:** None declared.

## References

[1] Bartos JA, Grunau B, Carlson C, Duval S, Ripeckyj A, Kalra R, et al Improved survival with extracorporeal cardiopulmonary resuscitation despite progressive metabolic derangement associated with prolonged resuscitation. Circulation 2020;141:877–886. 31896278 10.1161/CIRCULATIONAHA.119.042173 PMC7069385

[2] Yannopoulos D, Bartos J, Raveendran G, Walser E, Connett J, Murray TA, et al Advanced reperfusion strategies for patients with out-of-hospital cardiac arrest and refractory ventricular fibrillation (ARREST): a phase 2, single centre, open-label, randomised controlled trial. Lancet 2020;396:1807–1816. 33197396 10.1016/S0140-6736(20)32338-2 PMC7856571

[3] Belohlavek J, Yannopoulos D, Smalcova J, Rob D, Bartos J, Huptych M, et al Intraarrest transport, extracorporeal cardiopulmonary resuscitation, and early invasive management in refractory out-of-hospital cardiac arrest: an individual patient data pooled analysis of two randomised trials. EClinicalMedicine 2023;59:101988. 37197707 10.1016/j.eclinm.2023.101988 PMC10184044

[4] Berg RA, Nadkarni VM, Clark AE, et al Incidence and outcomes of cardiopulmonary resuscitation in PICUs. Crit Care Med 2016;44:798–808. 26646466 10.1097/CCM.0000000000001484 PMC4809365

[5] Federman M, Sutton RM, Reeder RW, Ahmed T, Bell MJ, Berg RA, et al Survival with favorable neurologic outcome and quality of cardiopulmonary resuscitation following in-hospital cardiac arrest in children with cardiac disease compared with noncardiac disease. Pediatr Crit Care Med 2024;25:4–14. 37678381 10.1097/PCC.0000000000003368 PMC10843749

[6] Conrad SA, Broman LM, Taccone FS, Lorusso R, Malfertheiner MV, Pappalardo F, et al The extracorporeal life support organization Maastricht treaty for nomenclature in extracorporeal life support. A position paper of the extracorporeal life support organization. Am J Respir Crit Care Med 2018;198:447–451. 29614239 10.1164/rccm.201710-2130CP PMC6118026

[7] Mensink HA, Desai A, Cvetkovic M, Davidson M, Hoskote A, O’Callaghan M, et al The approach to extracorporeal cardiopulmonary resuscitation (ECPR) in children. A narrative review by the paediatric ECPR working group of EuroELSO. Perfusion 2024;39:81S–94S. 38651582 10.1177/02676591241236139

[8] Lasa JJ, Rogers RS, Localio R, Shults J, Raymond T, Gaies M, et al Extracorporeal cardiopulmonary resuscitation (E-CPR) during pediatric in-hospital cardiopulmonary arrest is associated with improved survival to discharge: a report from the American Heart Association's get with the guidelines-resuscitation (GWTG-R) registry. Circulation 2016;133:165–176. 26635402 10.1161/CIRCULATIONAHA.115.016082 PMC4814337

[9] Ezad SM, Ryan M, Donker DW, Pappalardo F, Barrett N, Camporota L, et al Unloading the left ventricle in venoarterial ECMO: in whom, when, and how? Circulation 2023;147:1237–1250. 37068133 10.1161/CIRCULATIONAHA.122.062371 PMC10217772

[10] Eliet J, Gaudard P, Zeroual N, Rouvière P, Albat B, Mourad M, et al Effect of Impella during veno-arterial extracorporeal membrane oxygenation on pulmonary artery flow as assessed by end-tidal carbon dioxide. ASAIO J 2018;64:502–507. 28953197 10.1097/MAT.0000000000000662

[11] Schrage B, Becher PM, Bernhardt A, Bezerra H, Blankenberg S, Brunner S, et al Left ventricular unloading is associated with lower mortality in patients with cardiogenic shock treated with venoarterial extracorporeal membrane oxygenation: results from an International, Multicenter Cohort study. Circulation 2020;142:2095–2106. 33032450 10.1161/CIRCULATIONAHA.120.048792 PMC7688081

[12] Dimas VV, Morray BH, Kim DW, Almond CS, Shahanavaz S, Tume S, et al A multicenter study of the impella device for mechanical support of the systemic circulation in pediatric and adolescent patients. Catheter Cardiovasc Interv 2017;90:124–129. 28295963 10.1002/ccd.26973 PMC5511055

[13] Fiorelli F, Panoulas V. Impella as unloading strategy during VA-ECMO: systematic review and meta-analysis. Rev Cardiovasc Med 2021;22:1503–1511. 34957789 10.31083/j.rcm2204154

[14] Pappalardo F, Schulte C, Pieri M, Schrage B, Contri R, Soeffker G, et al Concomitant implantation of Impella® on top of veno-arterial extracorporeal membrane oxygenation may improve survival of patients with cardiogenic shock. Eur J Heart Fail 2017;19:404–412. 27709750 10.1002/ejhf.668

[15] Meani P, Lorusso R, Pappalardo F. ECPella: concept, physiology and clinical applications. J Cardiothorac Vasc Anesth 2022;36:557–566. 33642170 10.1053/j.jvca.2021.01.056

[16] Gaisendrees C, Djordjevic I, Sabashnikov A, Adler C, Eghbalzadeh K, Ivanov B, et al Impact of left ventricular unloading using a peripheral Impella®-pump in eCPR patients. Artif Organs 2022;46:451–459. 34516014 10.1111/aor.14067

